# Early-born neurons in type II neuroblast lineages establish a larval primordium and integrate into adult circuitry during central complex development in *Drosophila*

**DOI:** 10.1186/1749-8104-8-6

**Published:** 2013-04-23

**Authors:** Nadia Riebli, Gudrun Viktorin, Heinrich Reichert

**Affiliations:** 1 Biozentrum, University of Basel, Klingelbergstrasse 50, Basel, CH-4056, Switzerland

**Keywords:** Neuroblast, Lineage, Fan-shaped body, Primordium, Metamorphosis

## Abstract

**Background:**

The central complex is a multimodal information-processing center in the insect brain composed of thousands of neurons representing more than 50 neural types arranged in a stereotyped modular neuroarchitecture. In *Drosophila*, the development of the central complex begins in the larval stages when immature structures termed primordia are formed. However, the identity and origin of the neurons that form these primordia and, hence, the fate of these neurons during subsequent metamorphosis and in the adult brain, are unknown.

**Results:**

Here, we used two *pointed-Gal4* lines to identify the neural cells that form the primordium of the fan-shaped body, a major component of the *Drosophila* central complex. We found that these early-born primordium neurons are generated by four identified type II neuroblasts that amplify neurogenesis through intermediate progenitors, and we demonstrate that these neurons generate the fan-shaped body primordium during larval development in a highly specific manner. Moreover, we characterize the extensive growth and differentiation that these early-born primordium neurons undergo during metamorphosis in pupal stages and show that these neurons persist in the adult central complex, where they manifest layer-specific innervation of the mature fan-shaped body.

**Conclusions:**

Taken together, these findings indicate that early-born neurons from type II neuroblast lineages have dual roles in the development of a complex brain neuropile. During larval stages they contribute to the formation of a specific central complex primordium; during subsequent pupal development they undergo extensive growth and differentiation and integrate into the modular circuitry of the adult brain central complex.

## Background

The highly complex circuitry of the *Drosophila* central brain is established in two developmental steps. The first step takes place during embryogenesis and gives rise to the relatively simple brain of the larva; the second step takes place during postembryonic larval and pupal development and results in the formation of the much more complex mature brain of the adult. Both the embryonically generated neural cell populations that make up the larval brain and the postembryonically generated neural cell populations that form the bulk of the adult brain develop from a set of approximately 100 neural stem-cell-like neuroblasts that derive from the cephalic neuroectoderm in the early embryo (reviewed in [1-4]).

During embryogenesis, these neuroblasts undergo a first series of stem-cell-like proliferative divisions in which they divide in an asymmetric manner to self-renew and produce secondary precursors, which give rise to postmitotic neural progeny (reviewed in [5-7]). At the end of embryogenesis, most neuroblasts enter a phase of quiescence, which separates the primary embryonic phase from the subsequent secondary postembryonic phase of neurogenesis [4,8,9]. Most neuroblasts in the central brain resume proliferation during early larval stages in response to factors involving nutritionally activated mitogens and glial-cell-dependent interactions [10,11]. The neural cells produced postembryonically during the larval phase differentiate in the subsequent pupal phase and contribute to the functional adult brain circuits [3,12-15].

Recent studies have shown that two different types of neuroblast lineages are present in the central brain of *Drosophila*[16-20]. Most central brain neuroblasts are type I, and they give rise to lineages that contain on average 100 to 150 cells. These type I neuroblasts divide asymmetrically to self-renew and produce a non-self-renewing progenitor called a ganglion mother cell (GMC), which only divides once to generate two postmitotic neural progeny, neurons or glial cells. In contrast, a small set of neuroblasts in the central brain (8 in each hemisphere) are type II and they give rise to remarkably large lineages averaging 450 cells. These type II neuroblasts divide asymmetrically to self-renew and produce a self-renewing secondary progenitor called an intermediate neural progenitor (INP). This INP acts as a transit amplifying cell which retains its ability to divide several more times and hence can give rise to numerous GMCs each of which divides to produce two neural cells. In consequence, a marked amplification of proliferation takes place in the type II neuroblast lineages.

Clonal analysis of the numerous neuronal progeny generated by each of the type II neuroblasts during postembryonic development indicates that the adult-specific secondary neurons in these lineages form complex and widespread longitudinal and commissural projections in the brain. Furthermore they also demonstrate that a subset of these secondary neurons form major arborizations in all of the compartments of the central complex neuropile [21-24]. The central complex of the adult brain is a prominent midline neuropile in the protocerebrum that is involved in multimodal information processing and memory as well as in coordination of motor control in locomotory behaviors [25-27]. It is composed of thousands of neurons representing more than 50 neural types that are arranged in a stereotyped modular neuroarchitecture in all insects [28-31]. In *Drosophila*, its principle component modules are referred to as the protocerebral bridge, the fan-shaped body, the ellipsoid body, and the (paired) noduli. All of these modular structures receive major innervation from neurons belonging to type II lineages. Indeed, the amplification of proliferation that characterizes type II neuroblast lineages is thought to be an important factor in generating the enormous number of central complex neurons during the relatively short period of postembryonic secondary neuron proliferation [24]. Interestingly, and possibly counterbalancing this amplified proliferation, elimination of excess neurons in these lineages through programmed cell death is required for the formation of correct innervation of the developing central complex neuropile [23].

The development of the central complex begins in larval stages, when immature structures termed primordial are formed in a symmetrical manner on either side of the brain midline [32]. In early larval stages, the brain midline is formed by numerous thin fascicles that make up the nascent supraesophageal commissure and additional fascicles are added during larval development [33]. At the third larval instar, but not at the preceding second larval instar, primordia of the immature fan-shaped body and the immature protocerebral bridge can be identified using global markers such as DN-cadherin [15,32]. However, the identity and origin of the neurons that form these primordia are unknown. Correspondingly, there is no information concerning the fate of these primordium-forming neurons during subsequent metamorphosis in pupal stages and in the adult brain. Thus, although the contribution of secondary neurons from type II lineages to the central complex neuropile has been investigated in some detail, nothing is known about the role of any type II neurons in the formation of the central complex primordia or in the subsequent development of these primordium neurons during central complex development and in the adult.

In the present work, we took advantage of the fact that the P1 isoform of the Ets transcription factor Pointed (Pnt) is specifically expressed in type II lineages [34]. We analyzed this type II lineage-specific expression using a *pntP1-Gal4* line to drive reporter gene expression in the late larval brain and observed that recently born adult-specific neurons as well as a set of early-born neurons that innervate the fan-shaped body primordia are labeled. Moreover, we used embryonically induced flip-out methods to demonstrate that the primordium-forming neural cells are generated by four identified type II lineages DM1, DM2, DM3, DM6 [21]. We then screened a collection of *pnt* enhancer-fragment *Gal4* lines [35] and show that the *R45F08-Gal4* line targets reporter gene expression specifically to this population of early-born neurons. Using this specific genetic access we found that these type II neurons generate the primordium of the central complex during larval development in a highly specific and exclusive manner, and we show that this bilaterally symmetric larval primordium already manifests the type of modular subdivision that characterizes the mature central complex. Finally we used the *R45F08-Gal4* driver to document the extensive growth and differentiation of these early-born primordium neurons that occurs during the development of the (unpaired) central complex in pupal stages. Furthermore we show that these neurons persist in the adult central complex where they manifest a layer-specific innervation of the mature fan-shaped body as well as innervation of the ellipsoid body and protocerebral bridge.

## Results

### Specific Gal4-based labeling identifies a bilaterally symmetric central complex primordium in the larval brain

**Figure 1:**
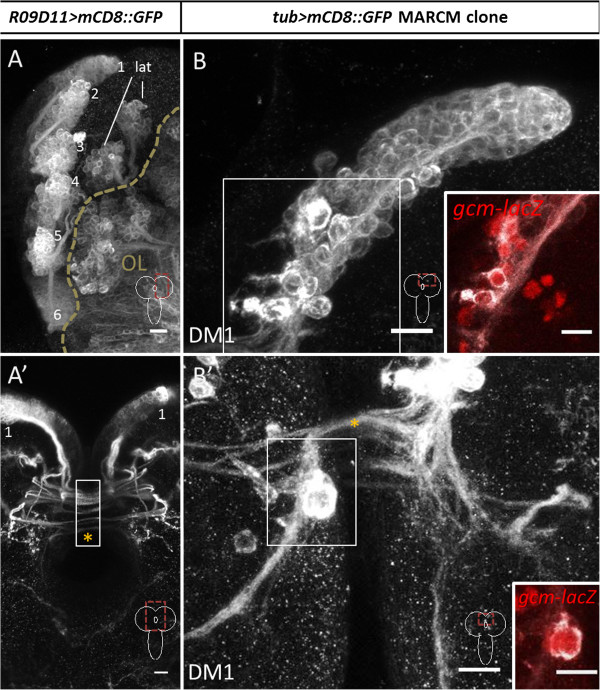
**Type II neuroblast lineages labeled by *R09D11-Gal4 *and mosaic analysis with a repressible cell marker (MARCM) in the late larval brain. (A,A’)***R09D11-Gal4* driven labeling of eight dorsomedial (DM) lineages and their commissural fascicles. DM1 to DM6 indicated with numbers; lat, lateral type II lineages. Z-projection of multiple adjacent optical sections. **(A)** shows one hemisphere, **(A’)** shows the midline regions of the larval brain with the commissure indicated by orange asterisk in **(A’)** and part of the commissural region overexposed to show fiber tracts (white box in A’). **(B,B’)***tubulin-Gal4* driven MARCM-based labeling of DM1 lineage shows neurons and glial cells in a single neuroblast clone. Axonal fascicles from the DM1 clone cross the commissure (asterisk in **(B’)**). Z-projection of multiple optical sections. The slightly larger cells at the base of the lineage are glial cells expressing *gcm-lacZ* (insets in **(B,B’)**; red, gcm-lacZ). Scale bars, 10 μm.

Previous work has shown that specific Gal4 lines that drive expression in type II neuroblasts and/or their progeny can be used in combination with *UAS-GFP* reporters to identify the cellular constituents of these lineages during development. Several useful Gal4 lines of this type have been generated by fusing the cis-regulatory DNA from developmental control genes that act specifically in type II lineages to Gal4 [35,36]. The development of type II neuroblast lineages in larval stages has been monitored with the *R09D11-Gal4* line, which specifically labels INPs and a large set of their (recently-born) progeny [22]. (The *R09D11-Gal4* line represents a fusion to Gal4 of regulatory DNA from the *earmuff* gene that is expressed in mature INPs and is thought to maintain the restricted proliferative potential of these cells and prevent dedifferentiation of these intermediate progenitors to ectopic neuroblasts; [37].) Figure 1A shows the eight type II lineages in one hemisphere of the third larval instar brain as revealed by the *R09D11-Gal4* line. Six of these type II lineages are located near the midline in the dorsoposterior region of the hemisphere. Each of these six lineages has been individually identified and, based on their spatial arrangement, have been referred to as DM1 (most dorsal/rostral), DM2, DM3, DM4, DM5, and DM6 (most ventral) [21]. (Given the fact that the neuroblasts of the type II lineages are located posteriorly and are Asense-negative, the lineages are also referred to as PAN lineages; [17].) The corresponding names given to these lineages by Pereanu and Hartenstein [15] are presented in Table 1. A further set of two type II lineages are located more laterally in the hemisphere. In this study, we focused primarily on the DM1 to DM6 lineages since they can be identified individually and have been characterized in most detail. In the larval brain, the cell bodies of each of the DM lineages are clustered together and neurites from each cell cluster form common axon fascicles that enter the neuropile and then arborize in a complex manner that is characteristic for each identified DM lineage [21]. A common feature of all DM lineages in the larval brain is that a subset of their neurites form a complex set of crossed and uncrossed commissural fiber tracts revealed by *R09D11-Gal4* (Figure 1A’, asterisk).

**Table 1:** Annotation of type-II NB dorsomedial (DM) lineages by different groups

**Group and reference**	**Annotation**					
Bello *et al*. [16]	DM1	DM2	DM3	DM4	DM5	DM6
Pereanu and Hartenstein [15]	DPMm1	DPMpm1	DPMpm2	CM4	CM3	CM1

Although *R09D11-Gal4* labels many of the cells in the type II lineages, it is much more strongly expressed in late-born cells which are located more proximally in the cell body cluster with respect to the neuroblast than early-born cells that are located distal to the neuroblast and closer to the hemisphere neuropile. (In the neuroblast clones of the central brain, late-born (recently born) cell bodies remain located close to their neuroblast of origin and displace the early-born cell bodies that are the most remote from their neuroblast of origin near the neuropile. Thus, the cells in a lineage are clustered and arranged along a spatiotemporal gradient; see [3].) A more complete visualization of all of the secondary, adult-specific neurons in each DM neuroblast lineage is possible by using mosaic analysis with a repressible cell marker (MARCM) techniques [38]. Figure 1B shows the type of labeled DM clone that can be revealed through MARCM labeling by using a ubiquitously expressed *tubulin-Gal4* driver with a *UAS-mCD8::GFP* reporter if recombination is induced after larval hatching and clones are recovered at late third instar. In the labeled DM1 clone shown, the neural progeny of the DM1 neuroblast are arranged in a spatiotemporal structured array that extends towards the neuropile. Several larger cells derive from this type II neuroblast that are located at the distal end of the lineage. These cells are labeled by both *gcm-lacZ* (Figure 1B,B’, insets) and Repo, but not Elav, and are thus glial cells, not neurons [39]. The secondary neurons in this MARCM-labeled DM1 lineage initially form a common secondary axon tract (SAT) that enters the neuropile and then arborizes in a highly complex, DM1-specific manner giving rise to interhemispheric commissural tracts, as well as ipsilateral and contralateral ascending and descending tracts [21]. Figure 1B’ shows the commissural tracts that the DM1 lineage neurons form at the brain midline (as well as a labeled glial cell; see inset). As in the case for most other secondary neurons in the larval central brain, all of the commissural and longitudinal tracts comprise immature neurons that only form terminal dendritic/axonal arbors and synaptic neuropile during pupal development [3,21,32].

**Figure 2:**
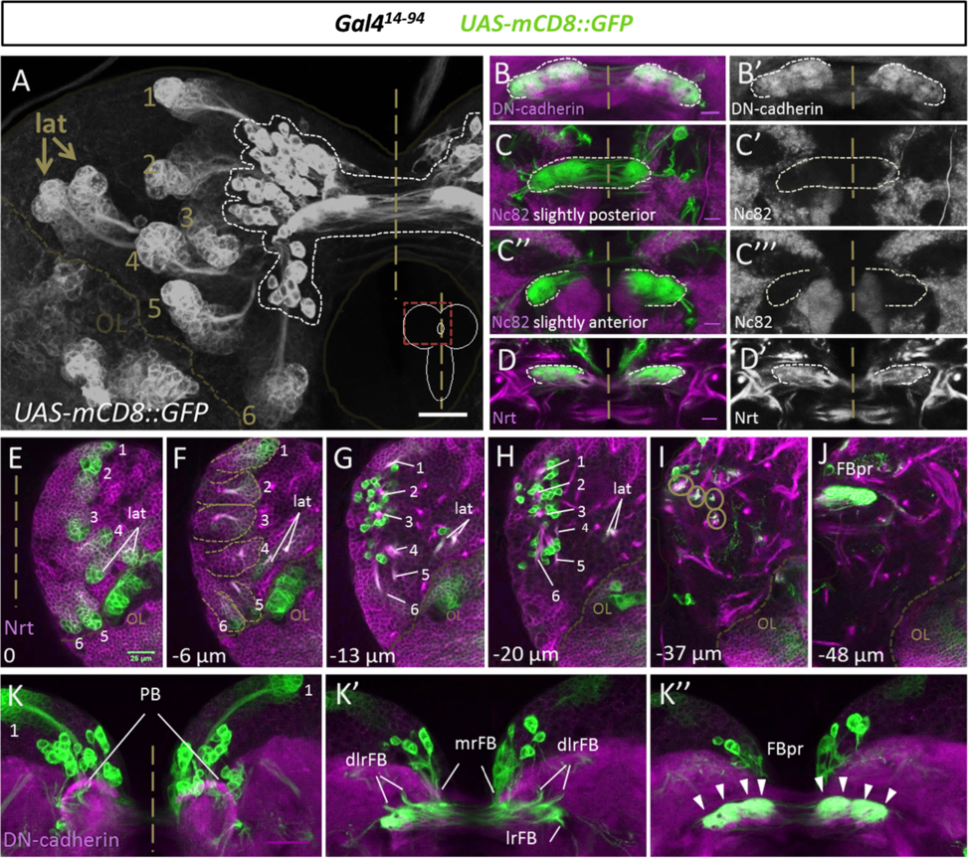
**Clusters of midline associated neurons and the fan-shaped body primordium are revealed by *Gal4***^***14-94***^**driven labeling of dorsomedial (DM) lineages in the late larval brain. (A)** Distal cell clusters corresponding to DM neuroblasts and their recently born progeny as well as a cluster of midline associated cells in close proximity to the fan-shaped body primordium are labeled by *Gal4*^*14-94*^. The dotted line indicates midline associated cells and primordium. DM1 to DM6 are indicated with numbers; lat, lateral type II lineages. Maximum projection of all stacks. **(B,B’)** The *Gal4*^*14-94*^-labeled fan-shaped body primordium (green) is colabeled with the neuropile marker DN-cadherin (magenta/white). **(C-C”’)** The *Gal4*^*14-94*^-labeled fan-shaped body primordium (green) is not colabeled with the synaptic marker Bruchpilot (magenta/white). **(D,D’)** The *Gal4*^*14-94*^-labeled fan-shaped body primordium (green) is colabeled with the membrane marker Neurotactin (magenta/white). **(E-J)** Single sections at different depths show the *GAL4*^*14-94*^-labeled cells of type II lineages (green) in a brain hemisphere colabeled for Neurotactin (magenta) revealing the relative position of the midline associated cells, the neurite fascicles of the DM lineages, and the fan-shaped body primordium (FBpr). DM1 to DM6 are indicated with numbers; lat, lateral type II lineages. Circles in **(I)** indicate neurite fascicles. **(K-K”)***GAL4*^*14-94*^-labeled cells and projections of type II lineages (green) at the midline colabeled for DN-cadherin (magenta). The two bilateral clusters of midline-associated cells **(K)** give rise to four neurite fascicles per hemisphere **(K’)**, which project into the fan-shaped body primordium (K”). The fan-shaped body primordium is composed of four subcompartments per hemisphere (arrowheads) **(K”)**. FBpr, fan-shaped body primordium; dlrFB, dorsolateral root of fan-shaped body; mrFB, medial root of fan-shaped body; lrFB, lateral root of fan-shaped body; nomenclature according to [40]. **(B-D’, K-K”)** are maximum intensity projections of few adjacent confocal slices. **(A)** and **(E-K”)** scale bars, 25 μm, (B-D’) scale bars, 10 μm.

Although both *R09D11-Gal4* labeling and *tubulin-Gal4* driven MARCM labeling allow visualization of the cell bodies and the axon fascicles that derive from these cell bodies, neither method reveals the central complex primordia (immature fan-shaped body, immature protocerebral bridge) that have been identified previously using global markers [32]. For this, other Gal4 lines are required. Recent work has shown that one isoform of the Ets transcription factor Pointed, PntP1, is specifically expressed in type II lineages and functions to promote the generation of mature INPs during larval development [34]. Correspondingly, a driver line in which Gal4 is integrated 347 base pairs before the transcription start site of a transcript that encodes PntP1, has been shown to drive reporter gene expression in type II neuroblasts, INPs, and a late-born subset of the secondary neurons specifically in all eight type II lineages of the late larval brain [34]; it also labels glial cells in these lineages (Additional file 1: Figure S1). (Like *R09D11-Gal4*, this Gal4 line also drives reporter gene expression in the optic lobe; this expression is not related to type II lineages in the central brain and, hence, is not considered further in this report.) Remarkably, we found that this Gal4 line, *Gal4*^*14-94*^, also very strongly labels a third cell type, neurons with large cell bodies, which are clustered near the distal ends of the DM lineages in each hemisphere and give rise to neurites that appear to project to a strongly-labeled structure near the brain commissure (Figure 2A). This labeled structure has all of the gross morphological features of the previously identified fan-shaped body primordium in the late larval brain, namely a bilaterally symmetrical, slightly curved bar-shaped structure that straddles both sides of the brain midline [32]. Moreover, the two halves of the structure appear to be interconnected by a plexus of commissural fibers, and each half of the structure is subdivided into (barely) discernible substructures. These observations suggest that the *Gal4*^*14-94*^ line may label the cells that contribute to the primordium of the fan-shaped body, in addition to labeling the type II neuroblasts, INPs, and their late-born progeny.

### The fan-shaped body primordium is innervated by early-born neurons of type II neuroblast lineages

To characterize the *Gal4*^*14-94*^-labeled midline structure in more detail, we first used immunocytochemical markers for DN-cadherin, Bruchpilot (NC82) and Neurotactin (BP106). The *Gal4*^*14-94*^-labeled midline structure was strongly immunoreactive for DN-cadherin and moderately immunoreactive for Neurotactin, however it was not immunoreactive for Bruchpilot (Figure 2B-D). Since the Bruchpilot protein is localized at the presynaptic active zone and is a marker for synaptic neuropile of differentiated neurons, this suggests that the labeled midline structure does not comprise synapses. Furthermore the lack of Bruchpilot staining suggests that the neurons building up the midline primordium are not yet differentiated. Previous studies have shown that DN-cadherin is expressed in primary neurons and their neural processes and synapses as well as in early secondary axons and filopodia, while Neurotactin labels the membranes and axon tracts of secondary axons [40,41]. Since Neurotactin and DN-cadherin are expressed in the fan-shaped body primordial structure but Bruchpilot expression is absent, this further suggests that this midline structure is composed of undifferentiated neural processes and filopodia. Indeed, the absence of synapses and the presence of as yet undifferentiated neural processes would be expected properties of the fan-shaped body primordium in the brain.

To study the anatomical relationship between the *Gal4*^*14-94*^-labeled cell bodies and the midline primordium structure further, we analyzed optical sections of different depth in late larval brains in which *Gal4*^*14-94*^ driven *UAS-mCD8::GFP* labeling was combined either with Neurotactin immunolabeling or with DN-cadherin immunolabeling (Figure 2E-K). We also compared *Gal4*^*14-94*^ directly with *R09D11-CD4::tdTom* reporter expression and confirmed that, while the two reporters overlap in the lineage proper close to the neuroblast (Additional file 2: Figure S2A,B), expression in the fan-shaped body primordium and the large neurons distal to the neuroblast is specific to *Gal4*^*14-94*^ (Additional file 2: Figure S2C,D). This analysis confirmed the notion that neurite-like processes from the *Gal4*^*14-94*^-labeled midline-associated cell clusters project to the labeled primordium structure. It also provided evidence for a fourfold modular organization of the primordium and of the innervating neurite tracts in each hemisphere. Moreover, it demonstrated that the labeled midline-associated cell bodies in each hemisphere are located adjacent to the nascent neuropile and clearly distant from the clusters of labeled neuroblasts, INPs and late-born secondary neurons in the type II lineages. Finally, this study revealed that the bilaterally symmetrical unfused primordium of the protocerebral bridge was also present at late third instar larval stage, but was not obviously labeled by *Gal4*^*14-94*^ or innervated by *Gal4*^*14-94*^-positive processes.

**Figure 3:**
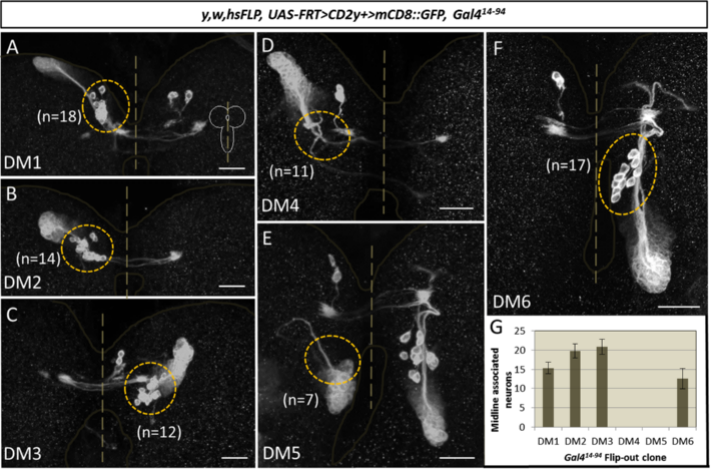
**The midline-associated cells are lineal descendants of four dorsomedial (DM) neuroblasts.** Embryonically induced flip-out clones showing that midline associated cells (cells in yellow dotted circles) are lineal descendants of four DM neuroblasts. **(A)** DM1 flip-out clone, **(B)** DM2 flip-out clone, **(C)** DM3 flip-out clone, **(D)** DM4 flip-out clone, **(E)** DM5 flip-out clone, **(F)** DM6 flip-out clone. Whereas DM1, DM2, DM3 and DM6 neuroblast clones invariably comprise between 10 and 23 midline associated cells, such cells were never found in DM4 and DM5 neuroblast clones. Dotted circles in **(A,B,C,F)** indicate midline associated cells, which are absent in **(D, E)**. **(G)** Average number of midline cells in DM flip-out clones, error bars are standard deviations. Scale bars, 25 μm.

**Figure 4:**
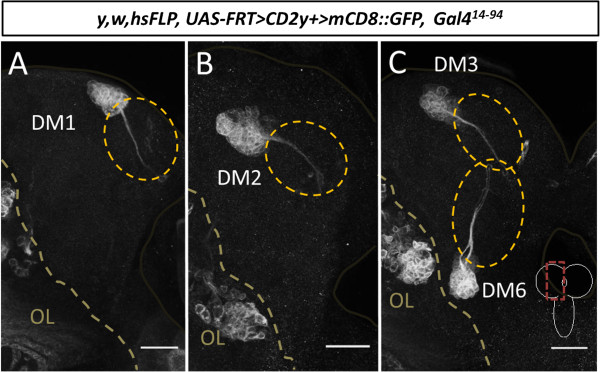
**The midline-associated cells are early-born lineal descendants of dorsomedial (DM) lineages DM1 to DM3 and DM6.** Flip-out clones induced at early L3 showing that no midline associated cells are produced by DM1 to DM3 and DM6 neuroblasts after this timepoint. However a substantial number of late secondary neurons is produced during the third larval instar. **(A)** DM1 flip-out clone, **(B)** DM2 flip-out clone, **(C)** DM3 and DM6 flip-out clone. Dotted circles in **(A-C)** indicate where midline associated cells would be located, but are absent. Scale bars, 25 μm.

In view of the spatial location of these labeled midline-associated cell bodies relative to the neuroblasts, INPs and late-born secondary neurons in the type II lineages, and considering the type II-specific labeling of the *Gal4*^*14-94*^ driver, we hypothesized that these cells might represent early-born neurons of type II lineages. To investigate this, we first determined if these midline-associated cells are lineal members of type II lineages. For this we used flip-out-based labeling of individual type II neuroblast clones in which the *Gal4*^*14-94*^ driver was coupled with a *UAS-FRT>CD2y*^*+*^*>mCD8::GFP* reporter and *hs-Flp*. For optimal labeling of early-born neurons, heat shock-Flp was induced in the early embryo (between 2 h and 4 h after egg laying) and clones were recovered at the late larval instar stage. (Flip-out labeling was used because early embryonic induction of MARCM-labeled neuroblast clones was not successful, as has also been reported elsewhere [42]). A minimum of seven *Gal4*^*14-94*^ driver labeled neuroblast clones in otherwise sparsely-labeled brains were recovered for each of the type II lineages DM1 to DM6. For DM1, DM2, DM3 and DM6, these clones invariably comprised both a group of cells consisting of the neuroblast together with its late-born progeny, and a set of more intensely labeled midline-associated neurons at the distal end of the lineages (Figure 3A-C,F-G). This indicates that the distal midline-associated cells are lineal descendants of the corresponding four type II neuroblasts, and together with the position of the cells distal to the neuroblast in a given clone implies that these are early-born lineal descendants. Moreover, the neurite tract emanating from the late-born neurons joins with and initially follows the neurite tract from the early-born neurons in each of these four DM lineages as might be expected for clonal descendants of the same neuroblast [15,41,43]. To confirm that these distal midline-associated cells are indeed early-born members of the lineages, we repeated the flip-out-based labeling of individual type II neuroblast clones but induced heat shock-Flp later at the early third larval instar. As expected, all of the clones recovered at the late third instar for DM1, DM2, DM3 and DM6 contained exclusively a group of cells consisting of the neuroblast together with its late-born progeny and never comprised the distal midline-associated neurons (Figure 4A-C).

Neuroblast clones of DM4 and DM5 type II lineages never contained the distally located set of intensely-labeled midline-associated neurons and were composed exclusively of the *Gal4*^*14-94*^-labeled neuroblast and its late-born progeny (Figure 3D,E). Quantification of the number of midline associated neurons in the DM1, DM2, DM3 and DM6 clones was carried out and indicated that each of the 4 neuroblast clones contained between 12 and 20 of these cells, resulting in total of approximately 70 midline-associated cells per hemisphere (Figure 3G). Since the total number of midline cells labeled by the *Gal4*^*14-94*^ driver corresponds to approximately 90 cells (91 ± 3; n = 4) per hemisphere, this indicates that most, but not all, of the *Gal4*^*14-94*^-labeled early-born cells are lineal descendants of the 4 type II neuroblast lineages DM1, DM2, DM3 and DM6. The remaining *Gal4*^*14-94*^-labeled early-born cells cannot be progeny of the two lateral type II lineages since all of the cells in these two lineages are positioned more laterally in the brain and show no spatial overlap with the midline cells. However, these midline neurons not generated by DM1 to DM3 and DM6 might be descendants of the DM4 and/or DM5 lineages that could be resistant to flip-out under the conditions used; due to technical constraints we were not able to determine their lineal origin with certainty.

Given that the primordium-forming neurons are derived from four different neuroblast lineages, we wondered if their processes in the primordium might be topologically organized. To investigate this, we generated flip-out clones using the *Gal4*^*14-94*^ driver as above, and recovered neuroblast clones or multicell clones from DM1 to DM3 and DM6 at the late larval stage. Up to three neuroblast/multicell clones were recovered in a given preparation. Analysis of their processes in the primordium indicates that there is in fact a topological order (Additional file 3: Figure S3). Thus, processes of the DM6 cells arborize most laterally on the ipsilateral side and most medially on the contralateral side of the primordium. Processes of the DM1 cells arborize most medially on the ipsilateral side and most laterally on the contralateral side of the primordium. Processes of the DM3 cells arborize adjacent to those of DM6 (medially adjacent on the ipsilateral side, laterally adjacent on the contralateral side). Processes of the DM2 cells arborize adjacent to those of DM1 (laterally adjacent on the ipsilateral side, medially adjacent on the contralateral side).

### A *pointed* enhancer fragment-Gal4 driver specifically labels neurons that exclusively innervate the fan-shaped body primordium

**Figure 5:**
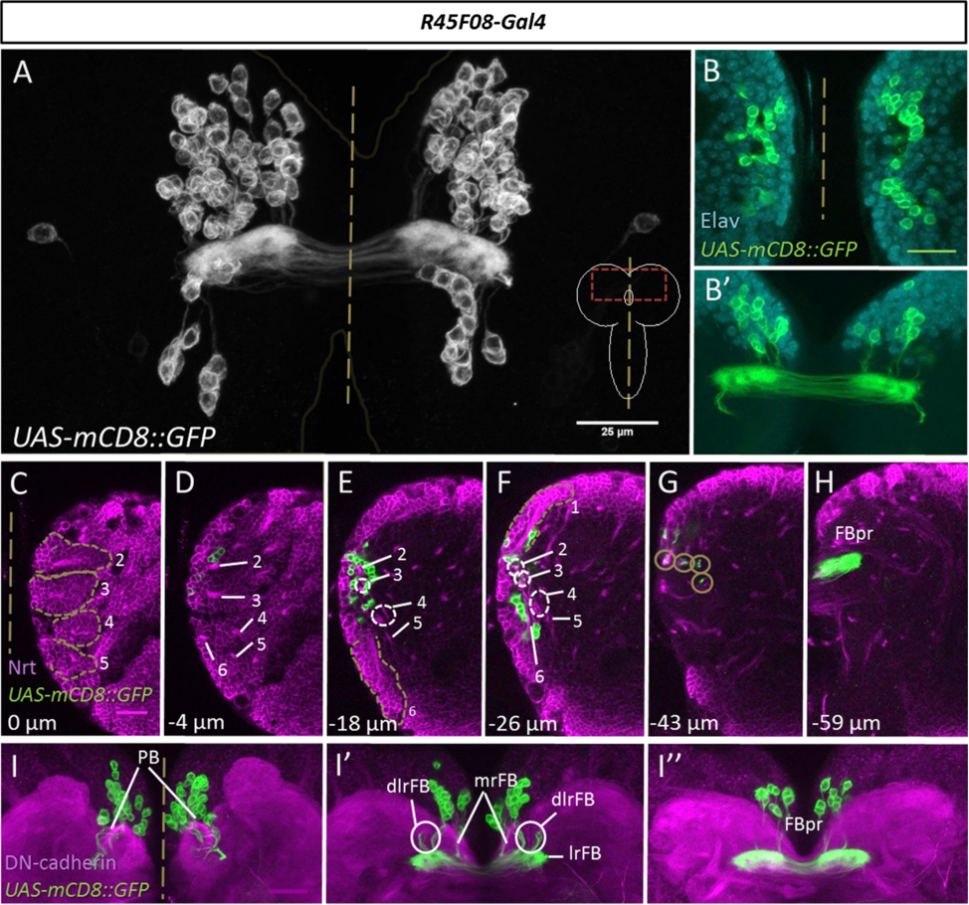
***R45F08-Gal4 *specifically labels the midline-associated neurons that innervate the fan-shaped body primordium in the late larval brain. (A)***R45F08-Gal4* labels two bilateral groups of midline-associated cells as well as the fan-shaped body primordium (FBpr). The number and position of the labeled cell bodies, which project their neurites into the fan-shaped body primordium, correspond to the number and position of the midline cells revealed by Gal4^14-94^ labeling (compare to Figure 2A; cells indicated by dotted line). No other neuronal structures are labeled implying that the labeled cell bodies give rise to the fan-shaped body primordium. **(B)** The *R45F08-Gal4*-labeled cells (green) are colabeled by the neuronal marker Elav (cyan). **(B,B’)** are two Z-projections taken at different focal planes. **(C-H)** Single sections taken at different depths show the *R45F08-Gal4*-labeled cells (green) in a brain hemisphere colabeled for Neurotactin (magenta) revealing the relative position of the primordium cell bodies **(D-F)**, their neurite fascicles **(F,G)**, and the fan-shaped body primordium (FBpr, **(H)**). Dorsomedial (DM) lineages DM1 to DM6 indicated with numbers. Circles in **(G)** indicate neurite fascicles. **(I-I”)***R45F08-Gal4*-labeled cells (green) at the midline of a brain colabeled for DN-cadherin (magenta). In each hemisphere, the group of labeled cell bodies gives rise to four neurite fascicles that project into the fan-shaped body primordium **(I”)**. One fascicle projects to the medial root of fan-shaped body (mrFB), one fascicle projects to the lateral root of the fan-shaped body (lrFB), and the remaining two fascicles project together to the dorsolateral root of the fan-shaped body (dlrFB); nomenclature according to [40]. **(I-I”)** are three Z-projections taken at different depths. Scale bars, 25 μm.

For a precise analysis of the development of the type II-derived midline-associated cells and their contribution to the central complex primordium, more specific genetic access that is restricted exclusively to these cells is required. To obtain this, we screened a set of driver lines in which different *pointed* cis-regulatory DNA fragments are fused to Gal4 [35]. Among these, we found that the *R45F08-Gal4* line drives reporter gene expression restricted exclusively to a set of cells that correspond to the type II-derived midline cells (Figure 5A). Thus, in the late larval brain *R45F08-Gal4* labels a set of approximately 90 (92 ± 3; n = 4) cell bodies in each hemisphere that are clustered near the midline and that project neurites into a strongly-labeled midline structure which has all of the morphological features of the fan-shaped body primordium. A comparison of larval brains labeled with *R45F08-Gal4* to larval brains labeled with *Gal4*^*14-94*^ shows that labeling with *R45F08-Gal4* is indeed restricted to the midline-associated primordium-forming cells; neither type II neuroblasts and INPs nor late-born neural cells and their neurite fascicles are labeled (compare Figure 2A with Figure 5A). The *R45F08-Gal4*-labeled cells are immunoreactive for the neuronal marker ELAV indicating that they are indeed neurons and not glial cells (Figure 5B). This was confirmed by the fact that none of the *R45F08-Gal4*-labeled cells showed anti-Repo immunoreactivity (data not shown).

To document the morphological relationship among the *R45F08-Gal4*-labeled cells, we analyzed optical sections of different depth in late larval brains in which *R45F08-Gal4* driven *UAS-mCD8::GFP* labeling was combined with either Neurotactin immunolabeling or with DN-cadherin immunolabeling (Figure 5C-I). This confirmed that neurite-like processes from the *R45F08-Gal4*-labeled cells project to the labeled fan-shaped body primordium. Moreover, it revealed the fourfold modular organization of the primordium and of the primordium-innervating neurite tracts in each hemisphere. Indeed, a comparison of *R45F08-Gal4*-labeled larval cells to *Gal4*^*14-94*^-labeled larval cells implies that all of the morphological features of the labeled fan-shaped body primordium are due to innervation by the *R45F08-Gal4*-labeled cells and, hence, do not result from innervation by other later-born secondary cells (compare Figures 2 and 5). Based on these findings, we concluded that the *R45F08-Gal4* line specifically labels the midline-associated type II lineage neurons that project their neurites exclusively into the fan-shaped body primordium in the larval brain, identifying these cells as the hitherto unknown primordium-forming neurons.

The remarkably specific labeling of the *R45F08-Gal4* line, which is restricted to the midline associated type II neurons, makes this driver line optimal for analyzing the subsequent developmental fate of these fan-shaped body primordium neurons during central complex formation and maturation in pupal stages and in the adult. This analysis shows that the primordium neurons undergo extensive growth and differentiation such that layer-specific innervation of the fan-shaped body is formed during metamorphosis, and this persists in the adult central complex. A spatiotemporal documentation of this differentiation process is presented below.

### The larval primordium neurons undergo extensive growth and differentiation and are integrated into the mature fan-shaped body of the adult brain

**Figure 6:**
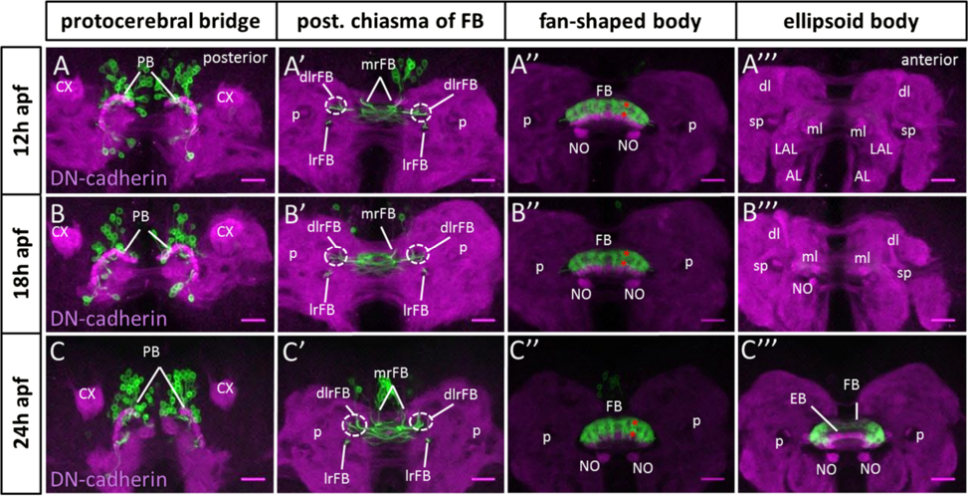
**Differentiation of larval fan-shaped body primordium-innervating neurons begins during early metamorphosis.** Spatiotemporal analysis of the *R45F08-Gal4* expressing cells and neurites as well as their innervation of developing central complex substructures during the first 24 h of metamorphosis. *R45F08-Gal4* labeling (green), DN-cadherin labeling of neuropile (magenta). Maximum intensity projections of few adjacent confocal slices taken at different depths. **(A-A”’)** At 12 h after puparium formation (apf) the *R45F08-Gal4*-labeled cells, located at the dorsomedial region of the brain cortex, bypass the protocerebral bridge and their fascicles project through midline chiasma and enter the developing fan-shaped body, whose two halves have fused, via the medial root of fan-shaped body (mrFB), the lateral root of the fan-shaped body (lrFB), and the dorsolateral root of the fan-shaped body (dlrFB). The labeled fibers arborize in the developing fan-shaped body and form eight (2 × 4) columnar innervation domains. Labeled fibers are not evident in other neuropiles. **(B-B”’)** At 18 h apf the *R45F08-Gal4*-labeled cells as well as their innervation of the developing fan-shaped body are largely unchanged as compared to 12 h apf. **(C-C”’)** At 24 h apf the *R45F08-Gal4*-labeled cells continue to project via midline chiasma and innervate eight columnar domains of the fan-shaped body, which has grown and become more bent. In addition sparse labeling is seen at the lateral edges of the developing (and yet unfused) ellipsoid body. In **(A”-C”)** asterisks indicate one labeled columnar domain in two layers of the fan-shaped body. AL, antennal lobe; CX, calyx; dlrFB, dorsolateral root of fan-shaped body; EB, ellipsoid body; FB, fan-shaped body; LAL, lateral accessory lobe; lrFB, lateral root of fan-shaped body; ml, medial lobe; mrFB, medial root of fan-shaped body; NO, noduli; PB, protocerebral bridge; p, peduncle; sp, spur of mushroom body. Neuroanatomical nomenclature according to [3,40]. Scale bars, 25 μm.

**Figure 7:**
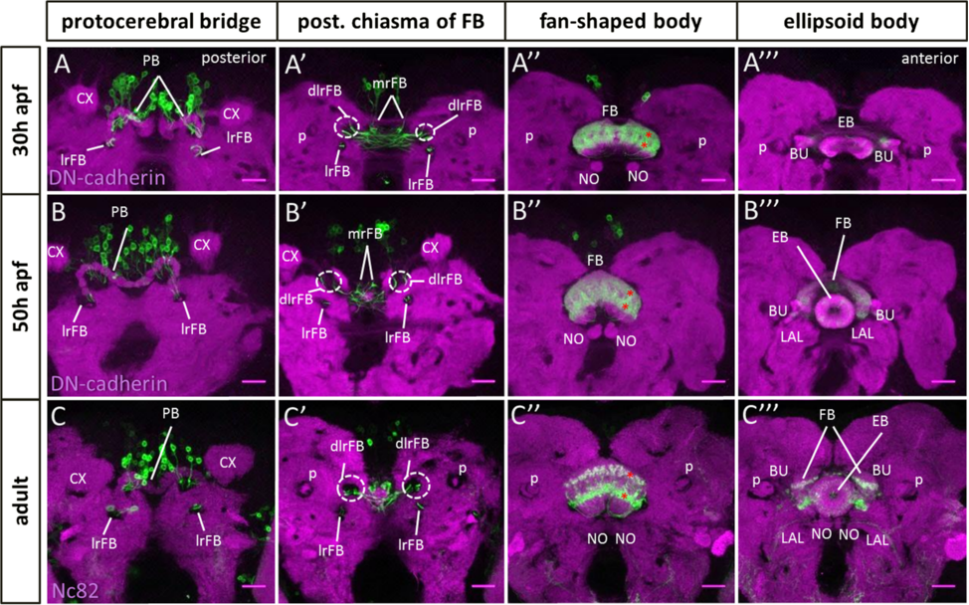
**Differentiation and integration of larval fan-shaped body primordium-innervating neurons into the mature central complex.** Spatiotemporal analysis of the *R45F08-Gal4* expressing cells and neurites as well as their innervation of developing central complex substructures at 30 h and 50 h of metamorphosis and in the adult. *R45F08-Gal4* labeling (green), **(A-B”’)** DN-cadherin labeling of neuropile (magenta), **(C-C”’)** Nc82 labeling of neuropile (magenta). Maximum intensity projections of few adjacent confocal slices taken at different depths. **(A-A”’)** At 30 h after puparium formation (apf) the *R45F08-Gal4*-labeled cells as well as their innervation of the developing fan-shaped body are similar to that observed at 24 h apf. **(B-B”’)** At 50 h apf, the innervation of the developing fan-shaped body by *R45F08-Gal4*-labeled cells continues to manifest eight columnar domains and labeled processes are also seen in the inner and outer layers of the ellipsoid body and in the bulbs. **(C-C”’)** In the adult the innervation of the mature fan-shaped body by *R45F08-Gal4*-labeled cells is largely restricted to two well separated layers, each of which is subdivided into the eight major columnar domains. Labeled processes remain visible in the ellipsoid body layers and in the bulbs. The noduli are not innervated by *R45F08-Gal4*-labeled cells at any stage. In **(A”-C”)** asterisks indicate one labeled columnar domain in two layers of the fan-shaped body. BU, bulbs, CX, calyx; dlrFB, dorsolateral root of fan-shaped body; EB, ellipsoid body; FB, fan-shaped body; LAL, lateral accessory lobe; lrFB, lateral root of fan-shaped body; mrFB, medial root of fan-shaped body; NO, noduli; PB, protocerebral bridge; p, peduncle. Neuroanatomical nomenclature according to [3,40]. Scale bars, 25 μm.

**Figure 8:**
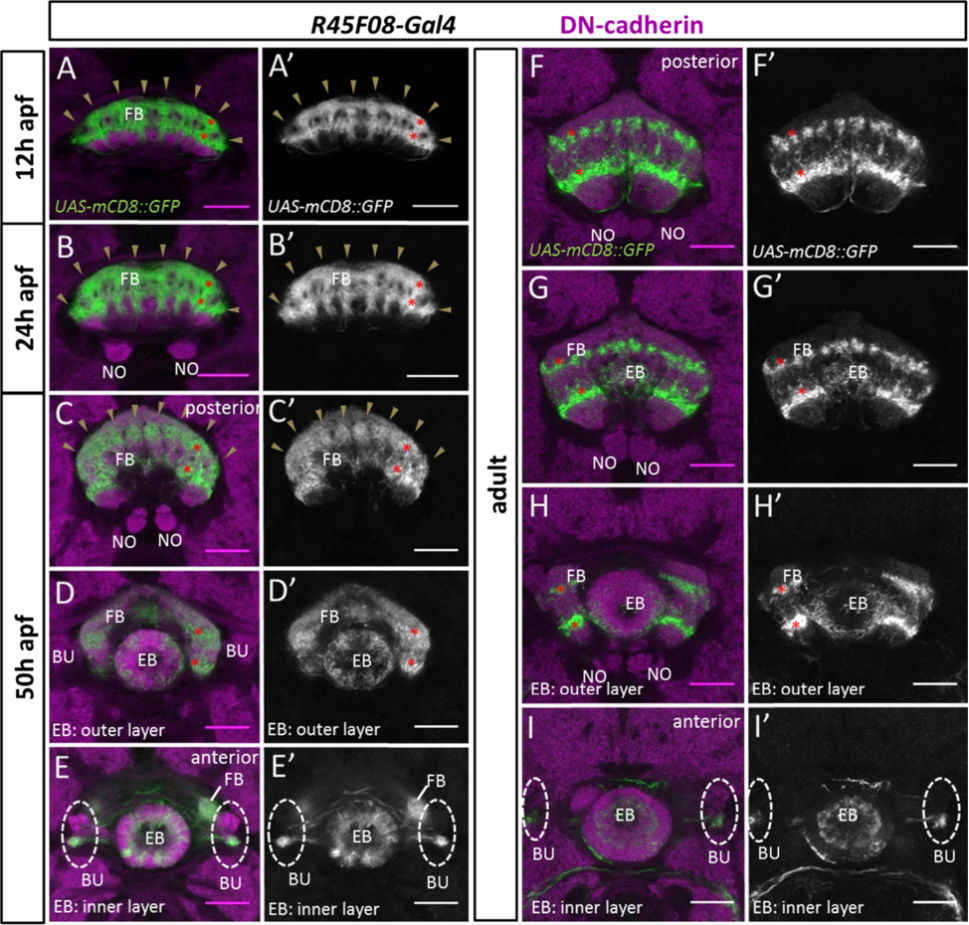
**Innervation of modular subdomains in the developing central complex neuropile by *R45F08-Gal4*-labeled neurons.** Spatiotemporal analysis of the modular innervation pattern of *R45F08-Gal4* expressing cells in the developing central complex during metamorphosis and in the adult. *R45F08-Gal4* labeling (green), DN-cadherin labeling of neuropile (magenta); all panels show single confocal sections. **(A,A’)** At 12 h after puparium formation (apf) *R45F08-Gal4*-labeled innervation of the developing fan-shaped body manifests eight columnar domains (arrowheads) arranged in two closely apposed horizontal layers. **(B,B’)** At 24 h apf *R45F08-Gal4*-labeled innervation of the developing fan-shaped body has grown but continues to show eight columnar domains (arrowheads) arranged in two closely apposed horizontal layers. **(C-E’)** At 50 h apf *R45F08-Gal4*-labeled innervation of the fan-shaped body has expanded further but is still seen in eight columnar domains (arrowheads) arranged in two horizontal layers. Labeled innervation is also seen in the inner and outer layer of the ellipsoid body, and innervation is seen in the ventral part of the bulbs. **(C,C’,D,D’,E,E’)** are taken at different focal planes along the A/P axis in the developing central complex. **(F-I’)** In the adult *R45F08-Gal4*-labeled innervation of the mature fan-shaped body is seen in two distinct layers that are clearly separated by unlabeled neuropile. Whereas in the ventral layer the eight columnar domains seem to have fused, in the dorsal layer the columns appear to be further subdivided into a 16-fold modular organization. Labeled innervation remains in the inner and outer layer of the ellipsoid body and the ventral part of the bulbs. **(F,F’,G,G’,H,H’,I,I’)** are taken at different focal planes along the A/P axis in the central complex. In **(A-D’,F-H’)** asterisks indicate one labeled columnar domain in two layers of the fan-shaped body. BU, bulbs; EB, ellipsoid body; FB, fan-shaped body; NO, noduli. Neuroanatomical nomenclature according to [3,40]. Scale bars, 25 μm.

In the following, the development of the larval primordium neurons was analyzed throughout early metamorphosis (Figure 6). Moreover, the primordium forming neurons and their arborizations were also analyzed during more advanced metamorphosis and into the adult (Figure 7). At 12 h after puparium formation (apf) the developing fan-shaped body has increased in size relative to the larval primordium, and its two halves have fused at the midline forming a seemingly unpaired midline structure that begins to bend ventrolaterally. Moreover, the protocerebral bridge primordium has grown larger and the noduli have become visible. (For a more detailed description of overall central complex development during metamorphosis, see [32].) At this stage, the *R45F08-Gal4*-labeled cell bodies of the primordium neurons are located at the dorsomedial midline of the central brain and project their neurites through a complex commissural chiasma into the developing fan-shaped body, where they form eight (2 × 4) prominent columns of innervation in two layers (Figures 6A and 8A). At 18 h apf, the overall development of the fan-shaped body, as well as its innervation by *R45F08-Gal4*-labeled processes, are largely similar to 12 h apf (Figure 6B). At 24 h apf, the fan-shaped body has further increased in size and become more bent. A slender, unfused ellipsoid body is visible rostral/ventral to the fan-shaped body, the protocerebral bridge has enlarged further, and the associated noduli have moved medially towards each other. At this stage, the *R45F08-Gal4*-labeled neurons continue to project through a midline plexus and their innervation of the fan-shaped body has grown but retains its modular arborization pattern in eight columns and two layers (Figure 6C and 8B). Very sparse *R45F08-Gal4*-labeled innervation of the ellipsoid body is seen at this stage (Figure 6C”’).

At 30 h apf, morphogenesis of the fan-shaped body is comparable to that seen at 24 h apf. The pattern of its innervation by *R45F08-Gal4*-labeled processes is also largely similar to that seen at 24 h apf. However, the ratio of labeled to unlabeled innervation of the fan-shaped body begins to be lower implying that other neurons are starting to contribute more, in relative terms, to the nascent neuropile of the fan-shaped body (Figure 7A). The first *R45F08-Gal4*-labeled processes are also visible in the bulbs, central complex-associated neuropile structures that appear around 30 h apf. Additional labeling is also apparent in the ellipsoid body (data not shown). At 50 h apf, the fan-shaped body has acquired the typical ‘fan-like’ form of the mature adult structure. The two halves of the protocerebral bridge as well as the two halves of the ellipsoid body have fused and the noduli have reached the midline. At this stage, the *R45F08-Gal4*-labeled cells continue to form eight columns in two layers in the fan-shaped body (Figures 7B and 8C-E). Labeled processes are also prominent in the inner and outer layers of the ellipsoid body as well as in the bulbs. In the adult brain, approximately 80 cells (76 ± 6; n = 3) per hemisphere are labeled by *R45F08-Gal4* as compared to approximately 90 cells per hemisphere that are *R45F08-Gal4*-labeled in the late larval brain. This indicates that most of the neurons that form the larval primordium survive and are integrated into the adult neuropile of the fan-shaped body. In the mature fan-shaped body of the adult, the *R45F08-Gal4*-labeled cells form two distinct layers that are clearly separated by relatively large domains of unlabeled neural processes that now represent to most of the neuropile (Figures 7C and 8F-I). The 8-fold repeated pattern shows a further subdivision suggestive of a 16-fold modular organization, notably in the upper layer of the fan-shaped body. Labeled processes remain present in the ellipsoid body layers and in the bulbs. It should be mentioned that very weak innervation of the protocerebral bridge by *R45F08-Gal4*-labeled cells is observed throughout pupal development but is not obvious in the adult.

**Figure 9:**
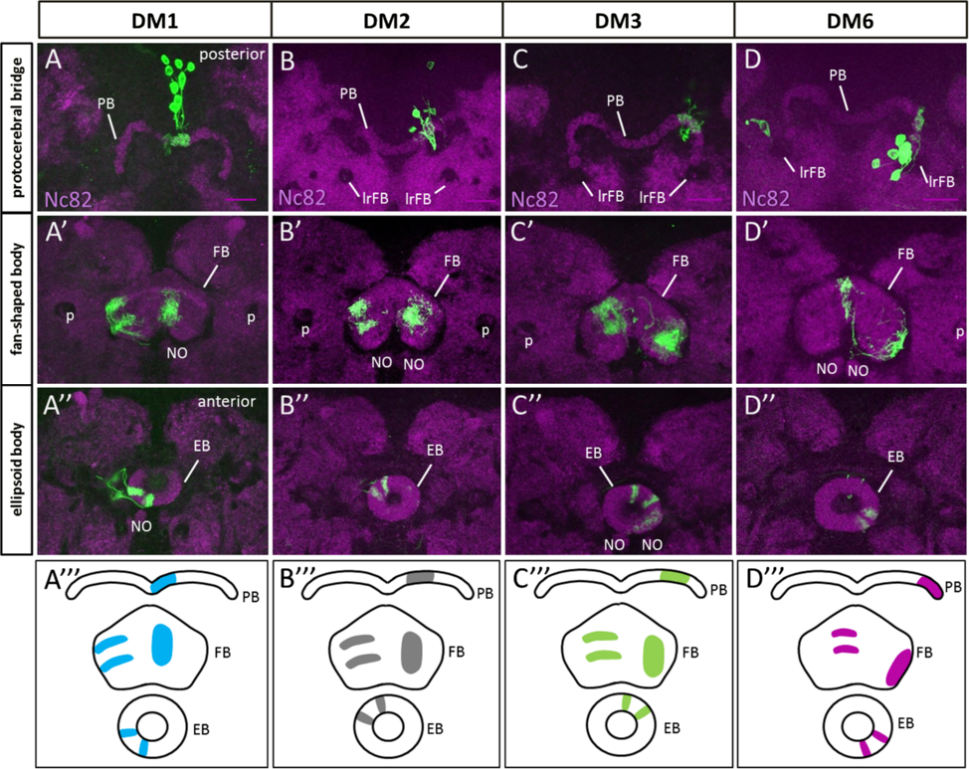
**The processes of *R45F08-Gal4*-labeled neurons display a topological organization in the central complex at 50 after puparium formation (apf).** Embryonically induced flip-out clones of dorsomedial (DM) lineages DM1 to DM3 and DM6 showing distinct arborization pattern by *R45F08-Gal4*-labeled neurons in the protocerebral bridge, the fan-shaped body and the ellipsoid body of the central complex. *R45F08-Gal4* flip-out clones labeling (green), Nc82 labeling of neuropile (magenta). Maximum intensity projections of few adjacent confocal slices taken at different depths. **(A-A”’)** DM1-derived midline associated cells projecting most medially on the ipsilateral side of the protocerebral bridge and the fan-shaped body and additionally innervating on the contralateral side of the fan-shaped body (most laterally) and the ellipsoid body. **(B-B”’)** DM2-derived midline associated cells projecting adjacent to DM1 more laterally on the ipsilateral side of the protocerebral bridge and the fan-shaped body and additionally innervating on the contralateral side of the fan-shaped body (medially adjacent to DM1) and the ellipsoid body. **(C-C”’)** DM3-derived midline associated cells projecting adjacent to DM2 more laterally on the ipsilateral side of the protocerebral bridge and the fan-shaped body and additionally innervating on the contralateral side of the fan-shaped body (medially adjacent to DM2). DM3 innervates the ellipsoid body on the ipsilateral side. **(D-D”’)** DM6-derived midline associated cells projecting most laterally on the ipsilateral side of the protocerebral bridge and the fan-shaped body and additionally innervating on the contralateral side of the fan-shaped body (most medially). DM6 innervates the ellipsoid body on the ipsilateral side. EB, ellipsoid body; FB, fan-shaped body; lrFB, lateral root of fan-shaped body; NO, noduli; PB, protocerebral bridge; p, peduncle. Neuroanatomical nomenclature according to [3,40]. Scale bars 25 μm.

Given the lineage-specific topological order of the early-born neuron processes in the fan-shaped body primordium of the larval brain, we wondered if this same topological order might be retained by these neurons in the developing fan-shaped body during pupal development. To investigate this, we generated flip-out clones using the *R45F08-Gal4* driver, and recovered neuroblast clones from DM1 to DM3 and DM6 at 50 h apf. Analysis of their arborizations in the protocerebral bridge, the fan-shaped body and the ellipsoid body indicates that there is indeed a topological order of these early-born neurons (Figure 9). Moreover, this lineage specific topological order is the same as that observed for the early-born neurons of the DM1 to DM3 and DM6 lineages in the larval primordium of the fan-shaped body. Thus, arbors of the DM6 cells arborize most laterally on the ipsilateral side and most medially on the contralateral side of the fan-shaped body. Processes of the DM1 cells arborize most medially on the ipsilateral side and most laterally on the contralateral side of the fan-shaped body. Processes of the DM3 cells arborize adjacent to those of DM6 (medially adjacent on the ipsilateral side, laterally adjacent on the contralateral side). Processes of the DM2 cells arborize adjacent to those of DM1 (laterally adjacent on the ipsilateral side, medially adjacent on the contralateral side). Topologically ordered innervation by these lineages is also seen in the protocerebral bridge and in the ellipsoid body at this 50 h apf stage (Figure 9).

Taken together, these findings indicate that the same *R45F08-Gal4*-labeled cells that form the bilaterally symmetric larval primordium of the fan-shaped body also contribute to the unpaired midline neuropile of the mature fan-shaped body. This occurs because the innervation of the fan-shaped body primordium that these cells form in larval stages, undergoes growth and differentiation during pupal development such that a highly patterned and restricted innervation of two layers of the adult fan-shaped body results.

## Discussion

In this report we used *pnt*-Gal4 lines to identify a specific subset of early-born neurons in four type II neuroblast lineages that contribute to a larval primordium of the central complex. Moreover, we demonstrated that this population of primordium neurons undergoes extensive growth and differentiation during metamorphosis, which results in a layer-specific innervation of the mature fan-shaped body of the adult. These findings reveal novel insights into the neural development of a larval brain primordium and well as into the integration of primordium neurons into the complex neuropile of the mature brain.

Previous studies have shown that a larval precursor of the fan-shaped body is present at the third larval instar stage [15,32]. However, neither the neurons that innervate this primordium nor the details of its structural organization were known. Using specific *pnt*-enhancer-Gal4 lines we have shown that neurons belonging to four identified pairs of type II neuroblast lineages innervate the larval fan-shaped body primordium. Remarkably, in the larval brain these neurons are involved exclusively in primordium innervation. The approximately 90 neurons identified by *R45F08-Gal4* labeling do not innervate any other part of the larval brain. This highly restricted innervation contrasts with the widespread innervation of many parts of the larval brain that other neurons in type II lineages form [21-23]. This, in turn, suggests that the primordium neurons represent a distinct, highly specified set of type II lineage cells that differ in their developmental genetic program from the other neurons in these lineages. It is tempting to speculate that the specific activation of a *pnt*-enhancer subunit in these cells, reflected by the highly specific nature of *R45F08-Gal4* labeling, is a consequence of this developmental genetic program.

Our data indicate that the neurons labeled by *R45F08-Gal4* correspond to early-born cells in each of their lineages. This implies that the neurons that are born first in the lineage also establish the precursor of the mature neuropile, which subsequently becomes innervated by that lineage during later development. Interestingly, the type II primordium neurons do not appear to form functional synapses during larval stages, since the neural processes in the larval primordium do not express the synaptic marker Bruchpilot. This implies that the primordium consists of immature neural processes from the type II early-born cells that, therefore, are not likely to be involved in larval brain function. Hence, we posit that the main function of the larval primordium is to serve as a scaffold for innervation and differentiation by later-born type II lineage neurons during metamorphosis in pupal stages.

It is noteworthy that the larval primordium is already subdivided into modular units, notably a fourfold subdivision of the hemiprimordium located in each hemisphere. This modularization of the primordium could serve as a structural basis for establishing the more complex (16-fold) columnar modularization of the mature fan-shaped body. The fourfold organization of the two hemiprimordia is reflected in the four innervating neurite tracts. Each of these four tracts derives from one of the four identified type II neuroblast lineages in each hemisphere. This modularization can be documented due to the highly restrictive labeling of the primordium neurons by *R45F08-Gal4*, which also makes it possible to deduce further organizational features of the primordium and its neural innervation. For example, the fact that the two hemiprimordia are interconnected by a prominent set of commissural fascicles indicates that neurons, whose cell bodies are located in one hemisphere, might innervate modular units on both sides of the primordium. Clonal labeling of the primordium-forming neurons indicates this is indeed the case. Moreover, there appears to be a lineage-specific topological order of projections from these early-born neurons to the primordium, and this topological order is retained by these neurons during subsequent fan-shaped body development. This general type of ‘bilateral’ innervation is typical of many of the numerous small-field neurons that connect small subdomains of compartments such as the fan-shaped body into an ordered array in the mature central complex [28,31].

A remarkable feature of the primordium neurons is that they persist throughout metamorphosis and become integrated into the mature central complex, where they form prominent layer-specific innervation of the fan-shaped body. This requires a marked growth and differentiation of these neurons’ innervation pattern from the non-layered innervation seen in the fan-shaped body primordium through successive stages of innervation expansion manifest as the central complex neuropile grows in early pupal stages, to the restricted pattern of innervation in two layers of fan-shaped body in the mature central complex at the end of metamorphosis. This restricted laminar innervation pattern in the adult suggests that these neurons might play specific functional roles in adult circuitry. Examples for the laminar regionalization of function are the neurons arborizing in specific horizontal strata of the fan-shaped body that have been shown to play important roles in visual memory (for example, [27]). Given the highly specific genetic access to the fan-shaped body primordium neurons provided by *R45F08-Gal4*, it should be feasible to investigate the role of these cells in behaving adult animals through targeted transgenic activation/inhibition and optogenetic methods. It is noteworthy that the number of (*R45F08-Gal4*-labeled) neurons that persist in the adult is comparable to that observed in the late larval brain. This implies that little, if any, cell death occurs in these early-born type II lineage neurons during metamorphosis. This contrasts with the pronounced degree of programmed cell death that has been reported to occur during postembryonic development in the overall ensemble of type II lineage cells [23], and provides further evidence for the notion that the early born differ in their developmental genetic program from other neurons in the type II lineages. The growth and differentiation of the primordium innervating neurons during metamorphosis is accompanied by a marked morphogenetic reorganization of the central complex itself. In addition to the overall growth of the neuropile due to increasing innervation and synapse formation, the most prominent aspect of this morphogenesis is the fusion of initially paired, bilaterally symmetrical hemiprimordia into an (apparently) unpaired midline neuropile, and this is seen both for the fan-shaped body and the protocerebral bridge.

## Conclusions

Taken together, these findings indicate that early-born neurons from type II lineages have dual roles in the development of complex brain neuropile. During larval stages they contribute to the formation of a specific central complex primordium. During subsequent pupal development they undergo extensive growth and differentiation and integrate into the modular circuitry of the central complex of the adult brain. Thus, in addition to generating a large number of structurally diverse neurons, some of which comprise the intrinsic neurons of the central complex, and giving rise to specific glial cells, some of which ensheath the neuropile components of the central complex [21,22,39], the type II neuroblasts also appear to generate neurons that establish a larval scaffold-like structure for the mature central complex. This provides further support for the notion that type II neuroblasts are remarkably multipotent neural stem cells that can generate the neural primordium, the mature neuronal cells, and the glial cells for one and the same complex brain structure.

## Methods

### Fly strains and genetics

Unless stated otherwise, fly stocks were obtained from the Bloomington Drosophila Stock Centre (Indiana University, Bloomington, IN, USA) and maintained on standard cornmeal medium at 25°C. For visualizing type-II neuroblast lineages *w*^*1118*^*, gcm-lacZ*^*rA87*^*, UAS-mCD8::GFP*^*LL5*^*/CyO,actin-gfp*^*JMR1*^; *R09D11-Gal4* (*GMR09D11*; [36]) recombined flies were crossed to *R09D11-Gal4*, *UAS-mCD8::GFP*^*LL6*^ flies. To generate wild type MARCM clones [38], we mated female *y, w, hs-Flp*^*1*^*; tubP-Gal4, UAS-mCD8::GFP*^*LL5*^*/CyO,actin-gfp*^*JMR1*^*; FRT82B, tub-Gal80*^*LL3*^[44] to *gcm-lacZ*^*rA87*^*/CyO, actin-gfp*^*JMR1*^; *FRT82B* males. Eggs were collected for 2 to 4 h, grown to first larval instar (22 to 30 h after egg laying), then heat shocked in a 37°C water bath (GFL 1083, Burgwedel, Germany) for 5 minutes. Larvae were then grown to late wandering third instar. The *Gal4*^*14-94*^[34] was kindly provided by the Jan lab (University of California, San Francisco, CA, USA). The *R45F08-Gal4* line is the *P{GMR45F08}attP2* enhancer-Gal4 line from Janelia Farm (Ashburn, VA, USA) [35]. For the spatiotemporal analysis the Gal4 lines were crossed to *y, w, UAS-Flp; UAS-Flp*^*JD1*^*; UAS-mCD8:GFP*^*LL5*^*/CyO, actin-gfp*^*JMR1*^; act5C>*>nlacZ,ry506 (act5C>*>nlacZ from [45]; flip-out-lacZ not relevant for this work). To analyze postlarval development, white prepupae were picked and kept at 25°C until they were dissected at the desired timepoints; 12 h pupae, however, were kept for 24 h at 18°C (where development takes twice as long as at 25°C). For analyzing late-born versus early-born type II neuroblast-derived cells, *UAS-mCD8::GFP*^*LL5*^*, Gal4*^*14-94*^ flies were crossed to *R09D11-CD4::tdTom*[46]. Flip-out clones were obtained by crossing *y, w, hs-Flp; UAS-FRT>CD2,y*^*+*^*>mCD8::GFP* (G. Struhl provided flies for publication in [47]) to the *Gal4*^*14-94*^ flies. Eggs were collected for 2 h and then heat shocked 2.5 to 4.5 h after egg laying in a 34°C water bath for 15 minutes. Late induced clones were heat shocked at 72 h to 79 h after egg laying in a 37°C water bath for 20 minutes. Then, larvae were grown to late wandering third instar.

### Immunolabeling

Larval brains were fixed and immunostained as described previously [39]. For all pupal and adult staining and for larval Elav staining, primary and secondary antibodies were incubated for 2 days at 4°C. For all other larval staining, primary antibodies were incubated overnight at 4°C and secondary antibodies were incubated for 3 h at room temperature. In Neurotactin (BP106) staining, a 5-minute methanol step was added after fixation and preliminary washings with PBS. Then 4 × 15 minute washings with PBS/0.5% Triton X-100 were performed before blocking. The following antibodies were used: rabbit anti-β-galactosidase 1:500 (55976, MP Biomedicals, Solon, OH, USA), chicken anti-GFP 1:1,000 (ab13970, Abcam, Cambridge, UK), mouse anti-Neurotactin 1:20 (BP106, Developmental Studies Hybridoma Bank (DSHB), Iowa city, Iowa, USA), rat anti-Elav 1:20 (7E8A10, DSHB), rabbit anti-Repo 1:400 (kindly provided by Veronica Rodrigues), rat anti-DN-cadherin 1:10 (DN-EX #8, DSHB), mouse anti-Nc82 1:20 (Bruchpilot, DSHB), rabbit anti-RFP 1:200 (ab62341, Abcam, Cambridge, UK). Alexa-conjugated secondary antibodies were used 1:300 (A11039, A11031, A21236, A21247, A11077, 21244, Molecular Probes, Eugene, OR, USA) and goat anti-mouse549 (Dylight™, KPL, Gaithersburg, MD, USA).

### Microscopy and image processing

All fluorescent images were recorded using a Leica TCS SP5 confocal microscope (Leica microsystems GmbH, Wetzlar, Germany). Optical sections ranged from 0.76 to 1 μm with a picture size of 1,024 × 1,024 pixels. Collected images were arranged and processed using Fiji [48]. Cell counts were performed with the Fiji plugin ‘cell counter’ (http://fiji.sc/Fiji). All adjustments were linear and were performed on whole images.

## Abbreviations

AL: antennal lobe; apf: after puparium formation; CX: calyx; dlrFB: dorsolateral root of fan-shaped body; DM: dorsomedial; DSHB: developmental studies hybridoma bank; EB: ellipsoid body; FB: fan-shaped body; FBpr: fan-shaped body primordium; GFP: green fluorescent protein; GMC: ganglion mother cell; INP: intermediate neural progenitor; LAL: lateral accessory lobe; lrFB: lateral root of fan-shaped body; MARCM: mosaic analysis with a repressible cell marker; ml: medial lobe; mrFB: medial root of fan-shaped body; NO: noduli; Pnt: pointed; p: peduncle; PB: protocerebral bridge; RFP: red fluorescent protein; sp: spur of mushroom body

## Competing interests

The authors declare that they have no competing interests.

## Authors’ contributions

NR and GV carried out all the experiments. HR conceptualized the project. NR, GV and HR analyzed the data and wrote the manuscript. All authors read and approved the final manuscript.

## Supplementary Material

Additional file 1: Figure S1. *Gal4*^*14-94*^ expressing neuroblast lineages contain neurons as well as glia cells. **(A-C)** Dorsomedial (DM)1 lineage cells of *Gal4*^*14-94*^ (green) at three different focal planes. The marker for differentiated glia (Repo) is in cyan and the marker for differentiated neurons (Elav) is in magenta. **(A)** Proximal to the considerably bigger neuroblast (arrow) there are closely associated, Elav-negative precursors (ganglion mother cells (GMCs) and intermediate neural progenitors (INPs), white dotted line), while many Elav-positive neurons are located more distally in the lineage (orange dotted line). **(B,C)** Closer to the commissure and even more distal to the neuroblast, the midline associated cells appear and show Elav expression (magenta arrowheads in **(B)** and **(C)**). At the level of the commissure (tracts crossing the commissure are indicated by red arrows in **(C)**) and even closer to the midline some glia are located (cyan arrowheads in **(C)**). Scale bars, 10 μm. Click here for file

Additional file 2: Figure S2. Midline associated cells and fan-shaped body primordium are revealed by *Gal4*^*14-94*^ labeling. Comparison of *Gal4*^*14-94*^ driven *mCD8::GFP* labeling and *R09D11-CD4::tdTom* labeling of type II neuroblast lineal cells in a late larval brain hemisphere. **(A-A”’,B-B”’,C-C”’,D-D”’)** Single confocal slices taken at four different depths of the same brain. *Gal4*^*14-94*^ labeling in green in **(A-D)** and in white in **(A”-D”)**, *R09D11-CD4::tdTom* expression in cyan in **(A’-D’)** and in white in **(A”’-D”’)**, and neurotactin labeling of neuropile in magenta. **(A-B”’)** Dorsomedial (DM) neuroblasts are labeled by *Gal4*^*14-94*^ and not by *R09D11-CD4::tdTom* but newly born cells located closely to the neuroblast are labeled by both *Gal4*^*14-94*^ and *R09D11-CD4::tdTom*. **(C)** At the level of the central brain neuropile, *Gal4*^*14-94*^ but not *R09D11-CD4::tdTom* labels the midline associated cells that are arranged around the fascicles of the DM lineages. **(D)** At the commissural midline the fan-shaped body primordium is labeled by *Gal4*^*14-94*^ but not by *R09D11-CD4::tdTom*. The numbers 2 to 6 correspond to lineages DM2 to DM6; DM1 is located in between the focal planes **(C)** and **(D)**. FBpr, fan-shaped body primordium; lat, lateral DMs. Scale bars, 25 μm. Click here for file

Additional file 3: Figure S3. The processes of midline associated neurons display a topological organization in the fan-shaped primordium at third larval instar. **(A)** Embryonically induced flip-out clones showing one dorsomedial DM6 neuroblast clone and two multicell clones of DM1 and DM3 and their fan-shaped primordium processes revealing a highly ordered arborization pattern within the forming central complex structure. Primordium-forming neurons are indicated by colored dots and arborization areas within the fan-shaped primordium by circles. Different colors are assigned to the different DM-derived cells and processes (magenta for DM6, green for DM3, blue for DM1 and dotted grey for prospective DM2-derived cells and arborization pattern). **(B)** The topological order of arborizations of the DM1 to DM3 and DM6-derived primordium-forming cells shown in a schematic. Different colors are assigned to the different DM-derived cells and processes (blue for DM1, grey for DM2, green for DM3 and magenta for DM6). Scale bar, 25 μm. Click here for file

## References

[B1] Urbach R Technau GM Neuroblast formation and patterning during early brain development in *Drosophila* Bioessays 2004 26 739 751 15221856 10.1002/bies.20062

[B2] Technau GM Berger C Urbach R Generation of cell diversity and segmental pattern in the embryonic central nervous system of *Drosophila* Dev Dyn 2006 235 861 869 16222713 10.1002/dvdy.20566

[B3] Hartenstein V Spindler S Pereanu W Fung S The development of the *Drosophila* larval brain Adv Exp Med Biol 2008 628 1 31 18683635 10.1007/978-0-387-78261-4_1

[B4] Egger B Chell JM Brand AH Insights into neural stem cell biology from flies Philos Trans R Soc Lond B Biol Sci 2008 363 39 56 17309865 10.1098/rstb.2006.2011 PMC2213715

[B5] Skeath JB Thor S Genetic control of *Drosophila* nerve cord development Curr Opin Neurobiol 2003 13 8 15 12593977 10.1016/s0959-4388(03)00007-2

[B6] Doe CQ Neural stem cells: balancing self-renewal with differentiation Development 2008 135 1575 1587 18356248 10.1242/dev.014977

[B7] Knoblich JA Mechanisms of asymmetric stem cell division Cell 2008 132 583 597 18295577 10.1016/j.cell.2008.02.007

[B8] Isshiki T Pearson B Holbrook S Doe CQ *Drosophila* neuroblasts sequentially express transcription factors which specify the temporal identity of their neuronal progeny Cell 2001 106 511 521 11525736 10.1016/s0092-8674(01)00465-2

[B9] Tsuji T Hasegawa E Isshiki T Neuroblast entry into quiescence is regulated intrinsically by the combined action of spatial Hox proteins and temporal identity factors Development 2008 135 3859 3869 18948419 10.1242/dev.025189

[B10] Chell JM Brand AH Nutrition-responsive glia control exit of neural stem cells from quiescence Cell 2010 143 1161 1173 21183078 10.1016/j.cell.2010.12.007 PMC3087489

[B11] Sousa-Nunes R Yee LL Gould AP Fat cells reactivate quiescent neuroblasts via TOR and glial insulin relays in *Drosophila* Nature 2011 471 508 512 21346761 10.1038/nature09867 PMC3146047

[B12] Truman JW Bate M Spatial and temporal patterns of neurogenesis in the central nervous system of *Drosophila melanogaster* Dev Biol 1988 125 145 157 3119399 10.1016/0012-1606(88)90067-x

[B13] Prokop A Technau GM The origin of postembryonic neuroblasts in the ventral nerve cord of *Drosophila melanogaster* Development 1991 111 79 88 1901786 10.1242/dev.111.1.79

[B14] Truman JW Schuppe H Shepherd D Williams DW Developmental architecture of adult-specific lineages in the ventral CNS of *Drosophila* Development 2004 131 5167 5184 15459108 10.1242/dev.01371

[B15] Pereanu W Hartenstein V Neural lineages of the *Drosophila* brain: a three-dimensional digital atlas of the pattern of lineage location and projection at the late larval stage J Neurosci 2006 26 5534 5553 16707805 10.1523/JNEUROSCI.4708-05.2006 PMC6675312

[B16] Bello BC Izergina N Caussinus E Reichert H Amplification of neural stem cell proliferation by intermediate progenitor cells in *Drosophila* brain development Neural Dev 2008 3 5 18284664 10.1186/1749-8104-3-5 PMC2265709

[B17] Bowman SK Rolland V Betschinger J Kinsey KA Emery G Knoblich JA The tumor suppressors Brat and Numb regulate transit-amplifying neuroblast lineages in *Drosophila* Dev Cell 2008 14 535 546 18342578 10.1016/j.devcel.2008.03.004 PMC2988195

[B18] Boone JQ Doe CQ Identification of *Drosophila* type II neuroblast lineages containing transit amplifying ganglion mother cells Dev Neurobiol 2008 68 1185 1195 18548484 10.1002/dneu.20648 PMC2804867

[B19] Reichert H *Drosophila* neural stem cells: cell cycle control of self-renewal, differentiation, and termination in brain development Results Probl Cell Differ 2011 53 529 546 21630158 10.1007/978-3-642-19065-0_21

[B20] Chang KC Wang C Wang H Balancing self-renewal and differentiation by asymmetric division: insights from brain tumor suppressors in *Drosophila* neural stem cells Bioessays 2012 34 301 310 22287225 10.1002/bies.201100090

[B21] Izergina N Balmer J Bello B Reichert H Postembryonic development of transit amplifying neuroblast lineages in the *Drosophila* brain Neural Dev 2009 4 44 20003348 10.1186/1749-8104-4-44 PMC2801669

[B22] Bayraktar OA Boone JQ Drummond ML Doe CQ *Drosophila* type II neuroblast lineages keep Prospero levels low to generate large clones that contribute to the adult brain central complex Neural Dev 2010 5 26 20920301 10.1186/1749-8104-5-26 PMC2958855

[B23] Jiang Y Reichert H Analysis of neural stem cell self-renewal and differentiation by transgenic RNAi in *Drosophila* Arch Biochem Biophys10.1016/j.abb.2012.08.003 22906721

[B24] Boyan GS Reichert H Mechanisms for complexity in the brain: generating the insect central complex Trends Neurosci 2011 34 247 257 21397959 10.1016/j.tins.2011.02.002

[B25] Strauss R Heisenberg M A higher control center of locomotor behavior in the *Drosophila* brain J Neurosci 1993 13 1852 1861 8478679 10.1523/JNEUROSCI.13-05-01852.1993 PMC6576564

[B26] Strauss R The central complex and the genetic dissection of locomotor behaviour Curr Opin Neurobiol 2002 12 633 638 12490252 10.1016/s0959-4388(02)00385-9

[B27] Liu G Seiler H Wen A Zars T Ito K Wolf R Heisenberg M Liu L Distinct memory traces for two visual features in the *Drosophila* brain Nature 2006 439 551 556 16452971 10.1038/nature04381

[B28] Hanesch U Fischbach KF Heisenberg M Neuronal architecture of the central complex in *Drosophila melanogaster* Cell Tissue Res 1989 257 343 366

[B29] Loesel R Nassel DR Strausfeld NJ Common design in a unique midline neuropil in the brains of arthropods Arthropod Struct Dev 2002 31 77 91 18088972 10.1016/S1467-8039(02)00017-8

[B30] Strausfeld NJ Brain organization and the origin of insects: an assessment Proc Biol Sci 2009 276 1929 1937 19324805 10.1098/rspb.2008.1471 PMC2677239

[B31] Young JM Armstrong JD Structure of the adult central complex in *Drosophila*: organization of distinct neuronal subsets J Comp Neurol 2010 518 1500 1524 20187142 10.1002/cne.22284

[B32] Young JM Armstrong JD Building the central complex in *Drosophila*: the generation and development of distinct neural subsets J Comp Neurol 2010 518 1525 1541 20187144 10.1002/cne.22285

[B33] Nassif C Noveen A Hartenstein V Early development of the *Drosophila* brain: III. The pattern of neuropile founder tracts during the larval period J Comp Neurol 2003 455 417 434 12508317 10.1002/cne.10482

[B34] Zhu S Barshow S Wildonger J Jan LY Jan YN Ets transcription factor Pointed promotes the generation of intermediate neural progenitors in *Drosophila* larval brains Proc Natl Acad Sci U S A 2011 108 20615 20620 22143802 10.1073/pnas.1118595109 PMC3251047

[B35] Jenett A Rubin GM Ngo TT Shepherd D Murphy C Dionne H Pfeiffer BD Cavallaro A Hall D Jeter J Iyer N Fetter D Hausenfluck JH Peng H Trautman ET Svirskas RR Myers EW Iwinski ZR Aso Y DePasquale GM Enos A Hulamm P Lam SC Li HH Laverty TR Long F Qu L Murphy SD Rokicki K Safford T A GAL4-driver line resource for *Drosophila* neurobiology Cell Rep 2012 2 991 1001 23063364 10.1016/j.celrep.2012.09.011 PMC3515021

[B36] Pfeiffer BD Jenett A Hammonds AS Ngo TT Misra S Murphy C Scully A Carlson JW Wan KH Laverty TR Mungall C Svirskas R Kadonaga JT Doe CQ Eisen MB Celniker SE Rubin GM Tools for neuroanatomy and neurogenetics in *Drosophila* Proc Natl Acad Sci U S A 2008 105 9715 9720 18621688 10.1073/pnas.0803697105 PMC2447866

[B37] Weng M Golden KL Lee CY dFezf/Earmuff maintains the restricted developmental potential of intermediate neural progenitors in *Drosophila* Dev Cell 2010 18 126 135 20152183 10.1016/j.devcel.2009.12.007 PMC6699514

[B38] Lee T Luo L Mosaic analysis with a repressible cell marker for studies of gene function in neuronal morphogenesis Neuron 1999 22 451 461 10197526 10.1016/s0896-6273(00)80701-1

[B39] Viktorin G Riebli N Popkova A Giangrande A Reichert H Multipotent neural stem cells generate glial cells of the central complex through transit amplifying intermediate progenitors in *Drosophila* brain development Dev Biol 2011 356 553 565 21708145 10.1016/j.ydbio.2011.06.013

[B40] Pereanu W Kumar A Jennett A Reichert H Hartenstein V Development-based compartmentalization of the *Drosophila* central brain J Comp Neurol 2010 518 2996 3023 20533357 10.1002/cne.22376 PMC2905803

[B41] Spindler SR Hartenstein V The *Drosophila* neural lineages: a model system to study brain development and circuitry Dev Genes Evol 2010 220 1 10 20306203 10.1007/s00427-010-0323-7 PMC2886914

[B42] Kunz T Kraft KF Technau GM Urbach R Origin of *Drosophila* mushroom body neuroblasts and generation of divergent embryonic lineages Development 2012 139 2510 2522 22675205 10.1242/dev.077883

[B43] Larsen C Shy D Spindler SR Fung S Pereanu W Younossi-Hartenstein A Hartenstein V Patterns of growth, axonal extension and axonal arborization of neuronal lineages in the developing *Drosophila* brain Dev Biol 2009 335 289 304 19538956 10.1016/j.ydbio.2009.06.015 PMC2785225

[B44] Bello BC Hirth F Gould AP A pulse of the *Drosophila* Hox protein Abdominal-A schedules the end of neural proliferation via neuroblast apoptosis Neuron 2003 37 209 219 12546817 10.1016/s0896-6273(02)01181-9

[B45] Struhl G Basler K Organizing activity of wingless protein in *Drosophila* Cell 1993 72 527 540 8440019 10.1016/0092-8674(93)90072-x

[B46] Han C Jan LY Jan YN Enhancer-driven membrane markers for analysis of nonautonomous mechanisms reveal neuron-glia interactions in *Drosophila* Proc Natl Acad Sci U S A 2011 108 9673 9678 21606367 10.1073/pnas.1106386108 PMC3111288

[B47] Wong AM Wang JW Axel R Spatial representation of the glomerular map in the *Drosophila* protocerebrum Cell 2002 109 229 241 12007409 10.1016/s0092-8674(02)00707-9

[B48] Schindelin J Fiji is just ImageJ - Batteries Included 6–7 November; 2008 Luxembourg: ImageJ User and Developer Conference

