# Supplementary figures and images for: Early-born neurons in type II neuroblast lineages establish a larval primordium and integrate into adult circuitry during central complex development in Drosophila
Source: Neural Dev. 2013 Apr 23;8:6. doi: 10.1186/1749-8104-8-6 (PMC3685605; doi:10.1186/1749-8104-8-6)

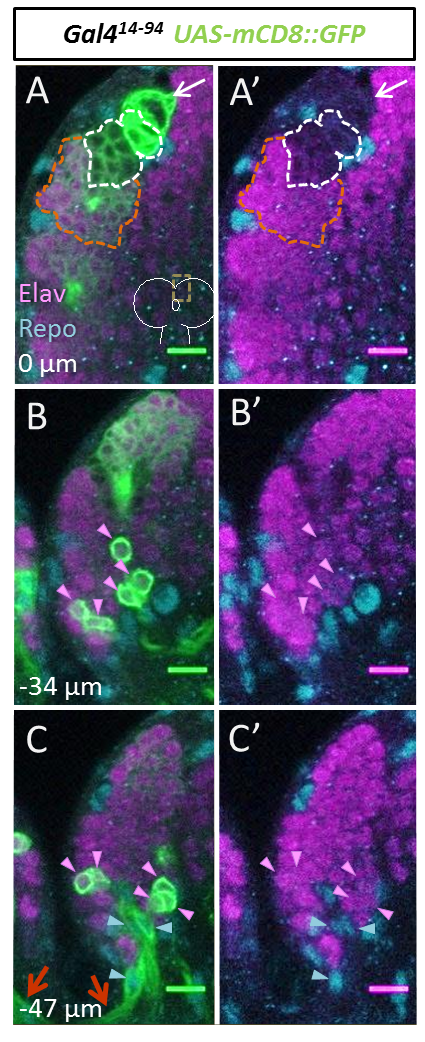

Supplement: Additional file 1: Figure S1. — Gal414-94 expressing neuroblast lineages contain neurons as well as glia cells. (A-C) Dorsomedial (DM)1 lineage cells of Gal414-94 (green) at three different focal planes. The marker for differentiated glia (Repo) is in cyan and the marker for differentiated neurons (Elav) is in magenta. (A) Proximal to the considerably bigger neuroblast (arrow) there are closely associated, Elav-negative precursors (ganglion mother cells (GMCs) and intermediate neural progenitors (INPs), white dotted line), while many Elav-positive neurons are located more distally in the lineage (orange dotted line). (B,C) Closer to the commissure and even more distal to the neuroblast, the midline associated cells appear and show Elav expression (magenta arrowheads in (B) and (C)). At the level of the commissure (tracts crossing the commissure are indicated by red arrows in (C)) and even closer to the midline some glia are located (cyan arrowheads in (C)). Scale bars, 10 μm. [file 1749-8104-8-6-S1.tiff]

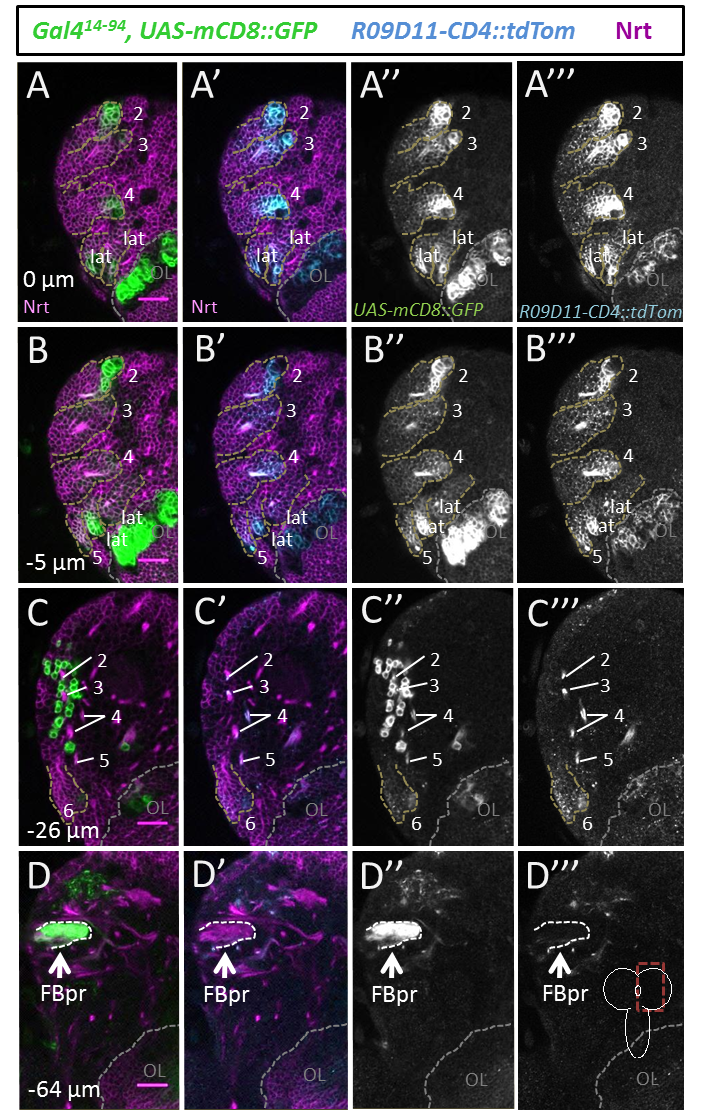

Supplement: Additional file 2: Figure S2. — Midline associated cells and fan-shaped body primordium are revealed by Gal414-94 labeling. Comparison of Gal414-94 driven mCD8::GFP labeling and R09D11-CD4::tdTom labeling of type II neuroblast lineal cells in a late larval brain hemisphere. (A-A”’,B-B”’,C-C”’,D-D”’) Single confocal slices taken at four different depths of the same brain. Gal414-94 labeling in green in (A-D) and in white in (A”-D”), R09D11-CD4::tdTom expression in cyan in (A’-D’) and in white in (A”’-D”’), and neurotactin labeling of neuropile in magenta. (A-B”’) Dorsomedial (DM) neuroblasts are labeled by Gal414-94 and not by R09D11-CD4::tdTom but newly born cells located closely to the neuroblast are labeled by both Gal414-94 and R09D11-CD4::tdTom. (C) At the level of the central brain neuropile, Gal414-94 but not R09D11-CD4::tdTom labels the midline associated cells that are arranged around the fascicles of the DM lineages. (D) At the commissural midline the fan-shaped body primordium is labeled by Gal414-94 but not by R09D11-CD4::tdTom. The numbers 2 to 6 correspond to lineages DM2 to DM6; DM1 is located in between the focal planes (C) and (D). FBpr, fan-shaped body primordium; lat, lateral DMs. Scale bars, 25 μm. [file 1749-8104-8-6-S2.tiff]

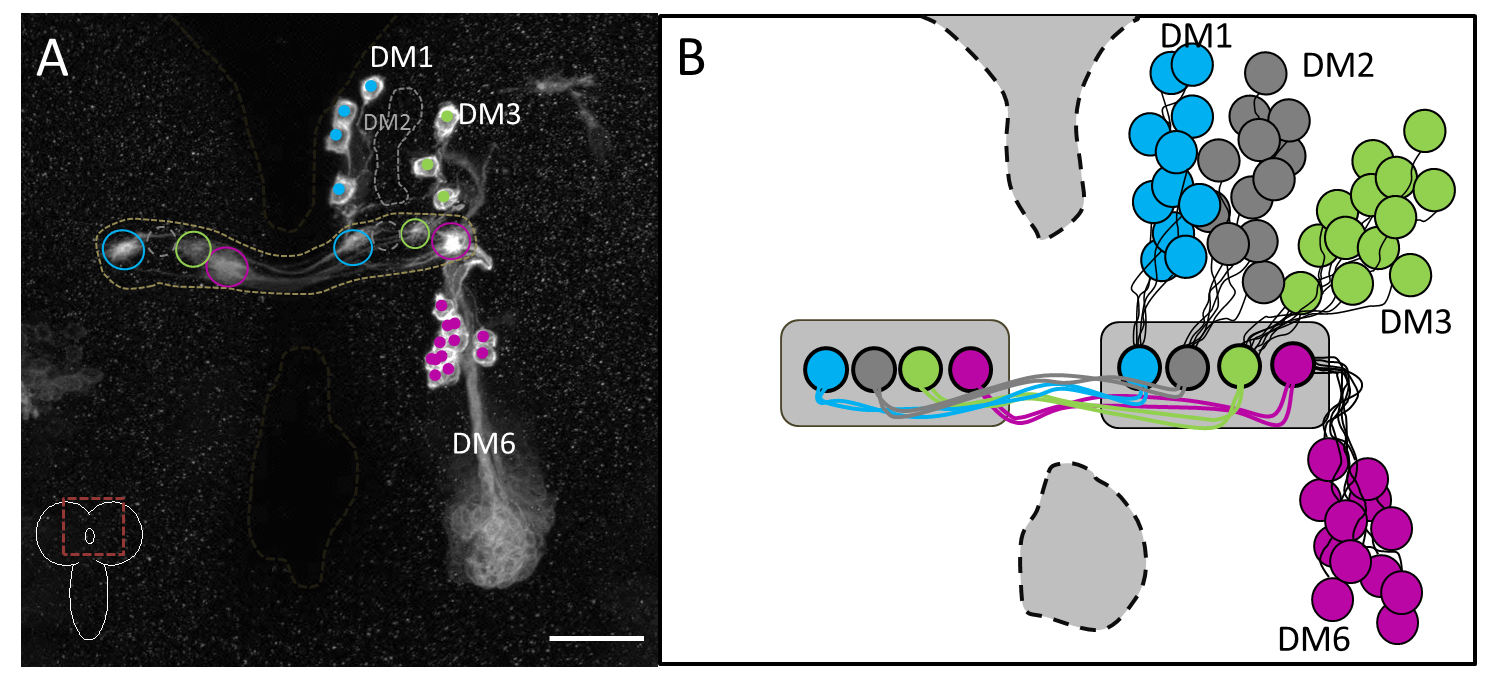

Supplement: Additional file 3: Figure S3. — The processes of midline associated neurons display a topological organization in the fan-shaped primordium at third larval instar. (A) Embryonically induced flip-out clones showing one dorsomedial DM6 neuroblast clone and two multicell clones of DM1 and DM3 and their fan-shaped primordium processes revealing a highly ordered arborization pattern within the forming central complex structure. Primordium-forming neurons are indicated by colored dots and arborization areas within the fan-shaped primordium by circles. Different colors are assigned to the different DM-derived cells and processes (magenta for DM6, green for DM3, blue for DM1 and dotted grey for prospective DM2-derived cells and arborization pattern). (B) The topological order of arborizations of the DM1 to DM3 and DM6-derived primordium-forming cells shown in a schematic. Different colors are assigned to the different DM-derived cells and processes (blue for DM1, grey for DM2, green for DM3 and magenta for DM6). Scale bar, 25 μm. [file 1749-8104-8-6-S3.tiff]
